# Microbial Communities in Pre-Columbian Coprolites

**DOI:** 10.1371/journal.pone.0065191

**Published:** 2013-06-05

**Authors:** Tasha M. Santiago-Rodriguez, Yvonne M. Narganes-Storde, Luis Chanlatte, Edwin Crespo-Torres, Gary A. Toranzos, Rafael Jimenez-Flores, Alice Hamrick, Raul J. Cano

**Affiliations:** 1 Department of Biology, University of Puerto Rico, San Juan, Puerto Rico, United States of America; 2 Center for Archaeological Research, University of Puerto Rico, San Juan, Puerto Rico, United States of America; 3 Dairy Products Technology Center, California Polytechnic State University, San Luis Obispo, California, United States of America; 4 Center for Applications in Biotechnology, California Polytechnic State University, San Luis Obispo, California, United States of America; University of Wisconsin, United States of America

## Abstract

The study of coprolites from earlier cultures represents a great opportunity to study an “unaltered” composition of the intestinal microbiota. To test this, pre-Columbian coprolites from two cultures, the Huecoid and Saladoid, were evaluated for the presence of DNA, proteins and lipids by cytochemical staining, human and/or dog-specific *Bacteroides* spp. by PCR, as well as bacteria, fungi and archaea using Terminal Restriction Fragment analyses. DNA, proteins and lipids, and human-specific *Bacteroides* DNA were detected in all coprolites. Multidimensional scaling analyses resulted in spatial arrangements of microbial profiles by culture, further supported by cluster analysis and ANOSIM. Differences between the microbial communities were positively correlated with culture, and SIMPER analysis indicated 68.8% dissimilarity between the Huecoid and Saladoid. Proteobacteria, Bacteroidetes and methanogens were found in all coprolite samples. Propionebacteria, *Shewanella* and lactic acid bacteria dominated in the Huecoid samples, while Acidobacteria, and peptococci were dominant in Saladoid samples. Yeasts, including *Candida albicans* and *Crypotococcus* spp. were found in all samples. Basidiomycetes were the most notable fungi in Huecoid samples while Ascomycetes predominated in Saladoid samples, suggesting differences in dietary habits. Our study provides an approach for the study of the microbial communities of coprolite samples from various cultures.

## Introduction

There is an increasing interest towards the intestinal microbiome as it can provide evidence of changes in host-microbe interactions. However, modern lifestyles may have a great impact on the composition of the intestinal microbiota [1]. One possible approach to study this effect is by collecting fecal samples of individuals of various geographical regions and cultures; yet, these reports are scarce due to limitations in the methods employed [2]. These studies consider fecal samples, not only from contemporary cultures, but also from individuals in isolated regions. It has also been suggested that the study of earlier cultures may represent a possible approach to study an “unaltered” composition of the intestinal microbiota [1]. Such is the case of pre-Columbian cultures, which were not affected by modern practices. The characterization of the intestinal microbiota of pre-Columbian humans may provide insights of microbial communities not affected by antibiotic usage and/or processed foods, for example. The Tainos represent a pre-Columbian culture that had a great cultural impact in modern societies in the Caribbean.

The Tainos were pre-Columbian inhabitants of the Bahamas, Greater Antilles and the northern Lesser Antilles. Prior to 1980, evidence supported that the Tainos were preceded by the Saladoid society, which in turn may have been constituted by two sub-cultures: the Cedrosan and Huecan Saladoid. The Saladoid society migrated from Venezuela during the last centuries of the pre-Christian era and the first of the Christian era, but differing archeological evidence has raised polemics about the Saladoid society. Specifically, during the 1970’s, archeologists Chanlatte and Narganes [3] found evidence of a pottery-making horticulturalist culture, even older than the Saladoid, that may have migrated from Bolivia and Colombia. The controversy began when it was proposed that this society, the Huecoid, was not a subgroup of the Saladoid, rather, a separate culture and an earlier migration of pottery-making horticulturalists [4], [5]. Differences between the Saladoid and Huecoid cultures so far are based on archeological evidence. For instance, unlike the Saladoids, the Huecoid culture did not paint their ceramic, there is no evidence of human burials in the Huecoid society, Saladoid and Huecoid houses are positioned differently and materials used to make tools differ between both societies. Saladoid and Huecoid archeological sites are characterized by the presence of animal remains. Extinct rodents have been found in the Saladoid archeological sites, but this culture is characterized by the presence of marine and fresh water turtles and bivalves, which were consumed. Animal remains such as rodents, iguanas, land snails and birds have been identified in the Huecoid deposits, and were consumed as well. Both cultures consumed mangrove land crabs, marine snails and gastropods [4], [5], [6]. Despite the notorious archeological evidence pointing out that the Saladoid and Huecoid are separate cultures, this is still not completely accepted by some members of the scientific community.

Currently, there is no other evidence of cultural differences between the Saladoid and Huecoid cultures. Insights of these differences may be studied through coprolites as diet is influenced by culture. Coprolites are desiccated fecal material which may provide information of cultural traditions, dietary habits and the status of the intestinal microbiota of an individual [7]. The amount, type and balance of the main dietary macronutrients (carbohydrates, proteins and fats) have a great impact on the intestinal microbiota. In addition, the gut microbiome harbor millions of genes, and thus can be one possible approach to distinguish individuals, even cultures [2]. The characterization of the intestinal microbiota of earlier cultures may serve as a baseline for studies of how modern lifestyles may influence intestinal microbial communities. In the present study, human coprolites were obtained from the Saladoid culture in the archeological sites of Sorcé in the island municipality of Vieques (Puerto Rico) and Tecla 1 in Guayanilla, Puerto Rico. Coprolite samples were also acquired from the Huecoid culture in the archeological site of Sorcé in Vieques. Coprolites were evaluated in terms of their source (human vs. animal), the presence of “informative” DNA, proteins and lipids and profiles of the intestinal microbiota of both groups were obtained as well.

## Results

### Bacterial, Fungal and Archaeal Identification

**Figure 1:**
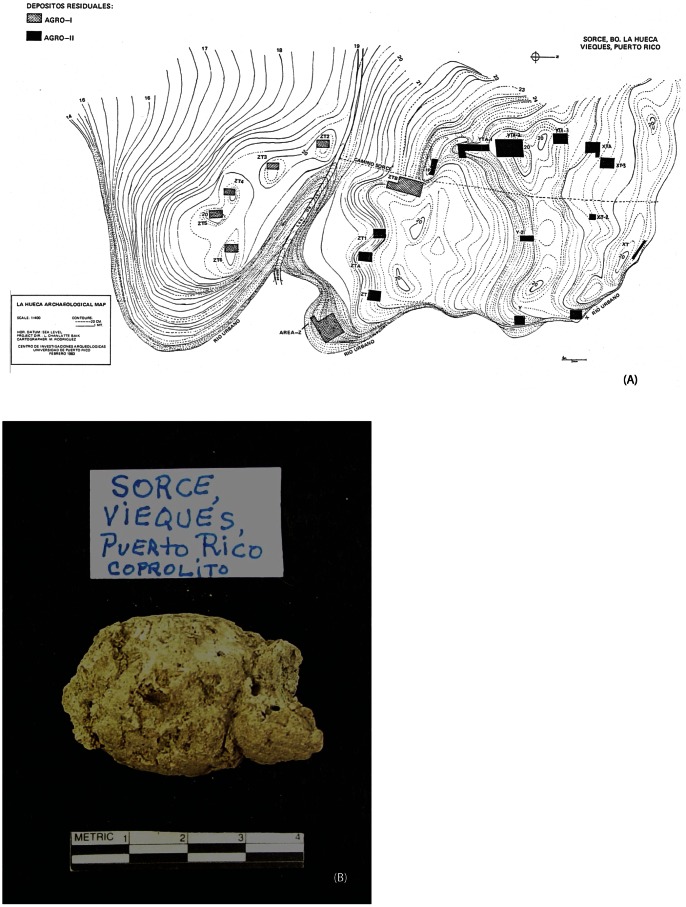
Archeological sites and coprolite samples in the present study. Panel (A) shows the deposits of the Saladoid and Huecoid groups in Vieques, Puerto Rico, from which the coprolite samples were collected. (B) Representative coprolite from the Sorcé, Vieques archaeological site.

**Figure 2:**
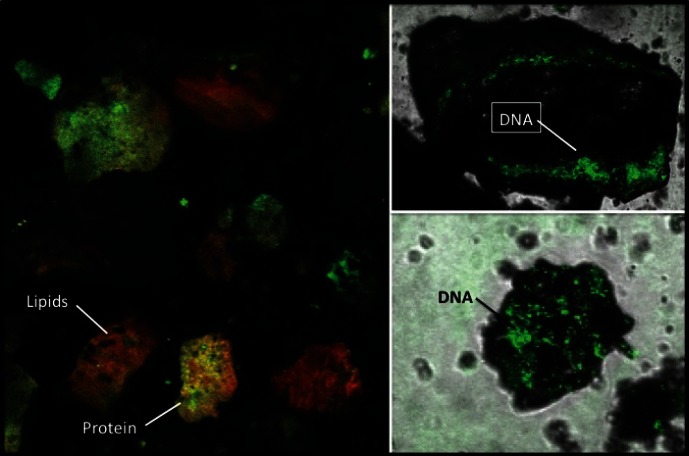
Presence of DNA, proteins and lipids in the coprolite samples. Detection of the macromolecules was determined using specific cytochemical staining.

Four coprolites originating from the archeological site of Sorcé in the island of Vieques (**Figure 1**) and one from the archeological site of Tecla 1 in Guayanilla, in south central Puerto Rico (**Table 1** **)** [8], were subjected to cytochemical stainining for the presence of macromolecules. DNA, proteins and lipids were successfully detected in all the coprolite samples by cytochemical staining (**Figure 2**), indicating the presence of analyzable DNA in the sample. The presence of human *Bacteroides* was evaluated by PCR and its presence detected in all five of the coprolite samples. None of the samples were positive for dog *Bacteroides*.

**Table 1:** Description of coprolites used in this study.

Deposit	Depth	Unit	Culture	Location	C-14 Dating
YTA-1	0.60cm.	I-5.	Saladoide	Vieques	335–395 A.D.
YTA-2	1.20mt.	I-24.	Saladoide	Vieques	230–385 A.D.
Z	0.40cm.	Z-X.	Huecoide	Vieques	470–600 A.D.
Z	1.80mt.	Z-W.	Huecoide	Vieques	Circa 180 A.D.
T-I-G	1.10mt.	M-64	Saladoide	Guayanilla	100 AC-300AD

**Figure 3:**
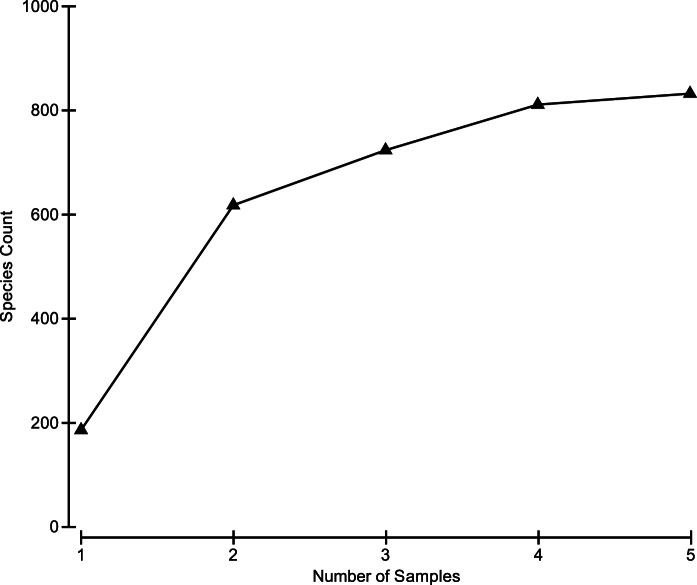
Cumulative number of unique TRF peaks accumulating with sample intensity. Values were calculated from the average of unique bands resulting from 50 permutations of random ordering.

The valuation of the data, as sufficient for the intended study was conducted by species accumulation plots. Leveling of the rarefaction curve indicated that bacteria, fungi and archaea detected by T-RFLP were sampled efficiently (**Figure 3**). The Saladoid coprolite samples exhibited a similarity percent of 31.16 and were characterized by the presence of bacteria of the genera *Haemophilus*, *Pseudoalteromonas*, *Corynebacterium*, *Bifidobacterium*, *Shewanella*, *Anoxybacillus*, *Mycoplasma* and *Desulfovibrio*. The average abundances, average similarities, contribution percents and cumulative percents of these bacterial genera are presented in **Table 2**. Fungi were also detected in the Saladoid coprolite samples and included the genera *Candida*, *Cryptococcus*, *Saccharomyces*, *Bullera*, *Penicillum*, *Melanconium*, *Absidia* and *Debaryomyces*. The average abundances, average similarities, contribution percents and cumulative percents of the fungal genera present in the Saladoid coprolite samples are also presented in **Table 2**. The Huecoid coprolite samples showed a higher similarity percent (58.17) compared to those of the Saladoid culture and were distinguished by bacteria of the genera *Bacteroides*, *Arthrobacter*, *Comamonas*, *Shewanella*, *Capnocytophaga*, *Actinobacillus*, *Acidobacteria* and *Acinetobacter*. The average abundances, average similarities, contribution percents and cumulative percents of the bacterial genera present in the Huecoid coprolite samples are shown in **Table 2**. Fungal genera in the Huecoid coprolite samples included *Cryptococcus*, *Candida*, *Melanconium*, *Saccharomyces*, *Penicillium*, *Leucosporidium*, *Bullera* and *Dictyoglomus*. Fungal average abundances, average similarities, contribution percents and cumulative percents of the bacterial genera present in the Huecoid coprolite samples are shown in **Table 2**. Database searches for archeal terminal restriction fragments (TRF) were unproductive as most of the taxa identified were either unculturable or unidentified archaeon. The few putatively-identified taxa *Methanobrevibacter* sp., *Methanosphaera* sp. and *Sulfolobus* sp. were found in all coprolites with no discriminatory power between archaeological sites.

**Table 2:** Similarity percentages for the Huecoid and Saladoid coprolites and the bacterial and fungal contributions.

Culture	TRF	Putative Taxon^1^	Ave. Abund.	Ave. Similar.	Contrib. %	Culture	TRF	Putative Taxon^1^	Ave. Abund.	Ave. Similar.	Contrib. %
**Saladoid (31.16% Similarity)**						**Huecoid (58.17% Similarity)**					
**Bacterial contributions**						**Bacterial contributions**					
364		*Haemophilus* sp.	179.32	5.05	16.21	366		*Bacteroides* sp.	208.69	3.76	6.47
365		*Pseudoalteromonas* sp.	217.41	4.08	13.1	367		*Arthrobacter* sp.	215.45	3.72	6.39
503		*Corynebacterium sp.*	193.82	3.78	12.14	365		*Comamonas* sp.	201.46	3.66	6.29
361		*Bifidobacterium sp.*	121.81	3.48	11.16	529		*Shewanella* sp.	185.37	3.44	5.92
529		*Shewanella* sp.	139.34	3.25	10.44	513		*Capnocytophaga* sp.	199.67	3.32	5.71
212		*Anoxybacillus* sp.	142.36	3.08	9.88	531		*Escherichia coli*.	177.56	3.27	5.63
528		*Mycoplasma* sp.	209.5	3	9.61	63		*Acidobacteria* sp.	170.25	3.13	5.38
93		*Desulfovibrio* sp.	94.46	2.8	8.97	526		*Acinetobacter sp.*	218.28	2.85	4.9
**Fungal contributions**						**Fungal contributions**					
79		*Candida* sp.	352.62	8.4	16.93	87		*Cryptococcus* sp.	327.66	6.91	17.95
87		*Cryptococcus* sp.	392.94	7.76	15.64	79		*Candida* sp.	336.22	4.87	12.67
82		*Saccharomyces* sp.	239	6.41	12.92	135		*Melanconium* sp.	249.34	4.71	12.24
85		*Bullera* sp.	210.02	5.73	11.55	82		*Saccharomyces* sp.	243.47	4.7	12.22
591		*Penicillium* sp.	185.83	4.4	8.86	591		*Penicillium* sp.	331.91	3.82	9.92
135		*Melanconium* sp.	259.85	4.31	8.68	506		*Leucosporidium* sp.	162.4	3.77	9.79
58		*Absidia* sp.	252.9	3.35	6.75	85		*Bullera* sp.	153.2	3.54	9.19
349		*Debaryomyces* sp.	97.48	3.3	6.66	585		*Dictyoglomus* sp.	198.44	3.45	8.96

Bacteria accounting for the dissimilarities between the Saladoid and Huecoid groups include *Anoxybacillus*, *Vibrio*, *Clostridium*, uncultured Actinobacteria, *Micrococcus*, *Lactobacillus*, *Alicyclobacillus*, *Geobacillus*, *Lysinibacillus* and *Fusobacterium* (Saladoid) and *Leuconostoc*, *Sulfitobacter*, *Brevibacterium*, *Dehalococcoides*, *Coprococcus*, *Cellulomonas*, *Xylella*, *Alicyclobacillus*, *Methylobacterium* and *Eubacterium* (Huecoid). Average dissimilarities, dissimilarities/SD, contribution and cumulative percents are shown in **Table 3**. Similarly, fungi responsible for dissimilarities between both cultures include *Melanconium*, *Debaryomyces*, *Candida*, unclassified Ascomycetes, *Venturia* and *Candida* (Saladoid), and *Leucoagaricus and Pleurotus* (Huecoid). Average dissimilarities, dissimilarities/SD, contribution and cumulative percents are shown in **Table 4**.

**Table 3:** SIMPER Analysis of Bacterial taxa impacting clustering of Huecoid and Saladoid Coprolite.

		Average Abundance					
TRF	Putative Taxon	Saladoid	Huecoid	Av. Diss.*	Diss./SD	Contrib.%	Cumul.%
212	*Anoxybacillus* sp.	142.36	0	2.05	3.12	3.12	3.12
527	*Vibrio* sp.	139.22	0	0.76	0.87	1.15	74.75
522	*Clostridium* sp.	121.90	0	0.65	0.87	0.99	89.37
361	Uncultured actinobacterium	121.81	0	1.75	19.05	2.67	11.54
505	*Micrococcus* sp.	120.94	0	0.69	0.87	1.05	80.13
519	*Lactobacillus* sp.	117.16	0	0.78	0.87	1.18	72.43
230	Uncultured acidobacterium	110.58	0	1.58	0.87	2.41	21.64
93	*Desulfovibrio* sp.	94.46	0	1.36	79.42	2.07	34.37
207	*Alicyclobacillus* sp.	92.6	0	0.68	0.87	1.04	84.32
243	*Geobacillus* sp.	78.66	0	1.13	0.87	1.71	43.55
512	*Lactobacillus* sp.	74.69	0	1.07	0.87	1.62	48.49
231	*Lysinibacillus* sp.	74.31	0	1.06	0.87	1.62	51.73
200	*Fusobacterium* sp.	63.33	0	0.92	0.87	1.39	60.73
63	*Leuconostoc* sp.	0	170.25	1.63	48.11	2.48	19.24
61	*Sulfitobacter* sp.	0	147.10	1.41	15.52	2.15	25.99
508	*Brevibacterium* sp.	0	144.15	1.38	5.00	2.10	30.23
497	*Dehalococcoides* sp.	0	111.63	1.07	0.87	1.62	50.11
178	*Coprococcus* sp.	0	105.94	1.01	0.87	1.54	56.37
371	*Cellulomonas* sp.	0	89.93	0.86	0.87	1.31	67.41
372	*Escherichia coli* sp.	0	88.89	0.85	0.87	1.29	68.70
206	*Alicyclobacillus* sp.	0	80.36	0.77	0.87	1.17	73.60
299	*Methylobacterium* sp.	0	71.70	0.69	0.87	1.05	82.23
376	*Eubacterium* sp.	0	66.97	0.65	0.87	0.98	90.35

* Total Average dissimilarity = 65.75.

**Table 4:** SIMPER Analysis of fungal taxa impacting clustering of Huecoid and Saladoid Coprolite.

		Average Abundance					
TRF	Putative Taxon	Saladoid	Huecoid	Av. Diss.	Diss./SD	Contrib.%	Cumul.%
136	*Melanconium* sp.	151.12	0	1.63	8.86	2.88	22.65
349	*Debaryomyces* sp.	97.48	0	1.72	13.68	3.03	10.91
519	*Candida* sp.	92.86	0	1.59	0.87	2.81	25.46
553	Unclassified Ascomycetes	92.72	0	1.67	0.87	2.96	16.85
75	*Venturia* sp.	89.87	0	1.58	17.68	2.79	31.04
133	Candida sp.	87.68	0	1.58	0.87	2.79	28.25
646	*Leucoagaricus* sp.	0	145.47	1.69	0.87	2.99	13.89
590	*Pleurotus* sp.	0	139.54	1.65	0.87	2.91	19.76

### Analyses of the Bacterial, Fungal and Archaeal Communities

**Figure 4:**
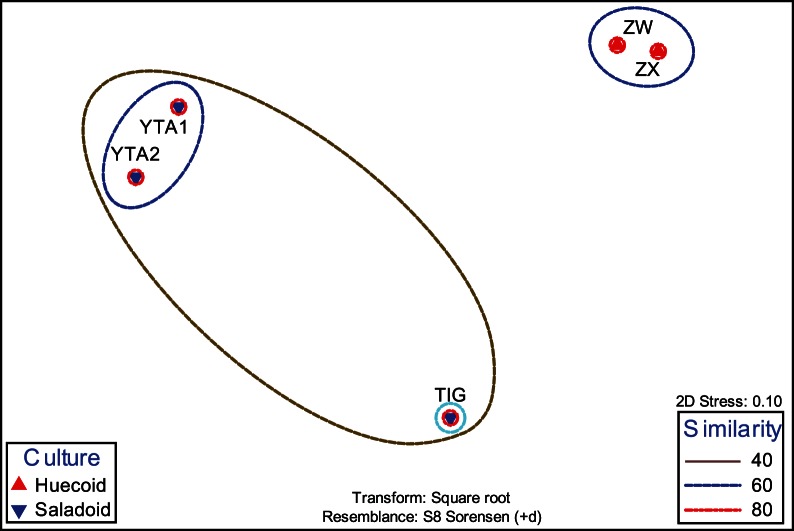
MDS plot of the microbial communities of the Saladoid and Huecoid societies in Vieques, Puerto Rico. Plot includes the Saladoid coprolite sample from Guayanilla, Puerto Rico as comparison.

**Figure 5:**
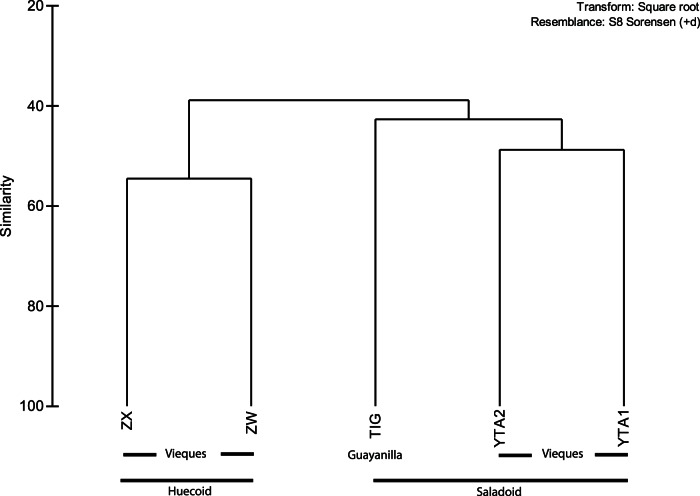
Dendrogram of the coprolite samples of the Saladoid and Huecoid cultures in Vieques, and the Saladoid culture in Guayanilla, Puerto Rico.

**Figure 6:**
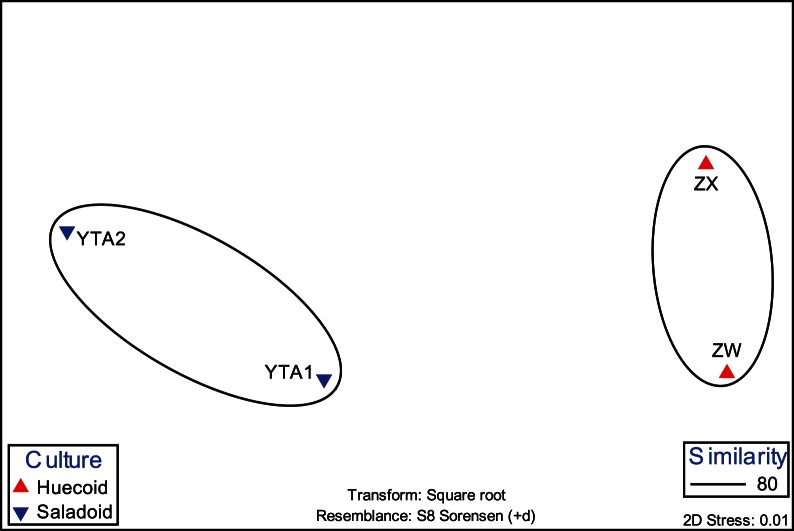
MDS analysis of the Saladoid and Huecoid coprolite samples from Vieques, Puerto Rico.

When the TRF area and height were analyzed for each enzyme, global R statistics revealed significant differences between the two archaeological sites (**Table 5**). These differences were more salient with bacteria and fungi. Archaeal T-RFLP analysis with the enzyme *Hha*I and fungal analysis with *Hpa*I indicated no significant difference between the two cultures. All other analyses with individual enzymes as well as the combined data for bacterial and fungal TRFs showed significant differences in cumulative R values. Microbial diversity, as estimated by standard indices of diversity, varied across the coprolite samples (**Table 6**). The MDS analyses showed an arrangement of the coprolite samples by culture (**Figure 4**). These results were further supported by the cluster analyses, in which the Saladoid and Huecoid cultures formed distinct clusters (**Figure 5**), and ANOSIM. When the Saladoid sample from Guayanilla was removed from the MDS analysis, a grouping of the coprolite samples by culture was still noticeable. Coprolite samples of the same culture exhibited similarities of 40% (**Figure 6**).

**Table 5:** R Statistics for Coprolites from Saladoid and Huecoid Archaeological Sites in Vieques.

Taxon	Enzyme	Cumulative R
Eubacteria	*Dpn*II	0.5
	*Hae*III	0.5
	*Hpa*I	0.3
	Combined	1.0
Fungi	*Aci*I	0.6
	*Hae*III	0.5
	*Hpa*I	0.0
	Combined	1.0
Archaea	*Hae*III	0.3
	*Hha*I	–0.3
	Combined	–0.3
All Taxa	Combined	1.0

**Table 6:** Diversity Statistics for all enzymes used for coprolites from Vieques archaeological sites.

Coprolite	Taxon	Enzyme(s)	Total TRF	Richness	Evenness	H’ (Shannon)	Simpson’s
ZW	Bacteria	*Dpn*II	26	1.810	0.939	3.060	0.941
		*Hae*III	27	3.057	0.986	3.250	0.959
		*Hha*I	31	3.489	0.993	3.410	0.966
		All eubacteria	84	16.589	0.985	5.079	0.993
	Fungi	*Hpa*I	25	2.839	0.981	3.159	0.955
		*Hae*III	21	2.397	0.973	2.963	0.943
		*Aci*I	3	0.276	0.785	0.863	0.497
		All fungi	49	5.195	0.966	3.761	0.972
	Archaea	*Hha*I	21	2.390	0.979	2.979	0.946
		*Hae*III	20	2.292	0.965	2.890	0.937
		All archaea	41	4.433	0.977	3.629	0.971
	ALL	All taxa	174	16.589	0.985	5.079	0.993
ZX	Eubacteria	*Dpn*II	31	2.171	0.965	3.313	0.958
		*Hae*III	25	2.833	0.986	3.173	0.956
		*Hha*I	31	3.496	0.988	3.393	0.965
		All eubacteria	87	19.355	0.988	5.261	0.994
	Fungi	*Hpa*I	27	3.061	0.982	3.237	0.958
		*Hae*III	23	2.627	0.970	3.040	0.947
		*Aci*I	27	3.062	0.981	3.233	0.958
		All fungi	77	7.956	0.983	4.272	0.985
	Archaea	*Hha*I	22	2.503	0.980	3.031	0.949
		*Hae*III	19	2.184	0.963	2.836	0.931
		All archaea	41	4.438	0.978	3.631	0.970
	ALL	All taxa	205	19.355	0.988	5.261	0.994
YTA1	Eubacteria	*Dpn*II	28	1.950	0.970	3.231	0.957
		*Hae*III	28	3.167	0.987	3.287	0.961
		*Hha*I	27	3.053	0.988	3.255	0.960
		All eubacteria	83	18.736	0.986	5.217	0.994
	Fungi	*Hpa*I	20	2.304	0.954	2.859	0.932
		*Hae*III	24	2.735	0.973	3.092	0.951
		*Aci*I	20	2.295	0.961	2.880	0.936
		All fungi	64	6.693	0.973	4.048	0.980
	Archaea	*Hha*I	27	3.064	0.980	3.230	0.958
		*Hae*III	24	2.736	0.973	3.093	0.950
		All archaea	51	5.470	0.981	3.857	0.977
	ALL	All taxa	198	18.736	0.986	5.217	0.994
YTA2	Eubacteria	*Dpn*II	29	2.027	0.960	3.233	0.955
		*Hae*III	26	2.948	0.983	3.204	0.957
		*Hha*I	28	3.162	0.990	3.298	0.962
		All eubacteria	83	16.469	0.986	5.079	0.993
	Fungi	*Hpa*I	21	2.394	0.976	2.970	0.945
		*Hae*III	19	2.168	0.974	2.868	0.939
		*Aci*I	21	2.408	0.963	2.933	0.939
		All fungi	61	6.369	0.979	4.023	0.980
	Archaea	*Hha*I	14	1.601	0.961	2.536	0.912
		*Hae*III	15	1.717	0.964	2.610	0.918
		All archaea	29	3.171	0.970	3.267	0.957
	ALL	All taxa	173	16.469	0.986	5.079	0.993

## Discussion

The present study evaluated the resident microbiota of coprolites as a source of information. As coprolites and other similar fossilized materials are subject to environmental contamination, the sample preparation, DNA extraction and PCR amplification for this study were conducted in areas designated for handling of such ancient materials and routinely monitored for extraneous DNA contamination. The information contained herein is predicated on the degree of preservation of macromolecules within the coprolite. In this study, we assessed the presence of macromolecules, including proteins and nucleic acids as a first step in the study to ensure that the information gathered represents what is contained in the coprolite and not what may come from environmental contamination. Cytochemical studies of coprolite material from the core of the coprolite indicated that proteins, lipids and DNA were detectable in the interior of the coprolites and therefore further analysis could ensue. Also, studies of proteins and lipids from human coprolites are still very limited [9]. The detection of proteins and lipids in the coprolite samples in the present study is very promising as these may provide nutritional and metabolic information.

The human origin of the coprolites was assessed, not only based on archaeological observations, but also on the detection of human-associated *Bacteroides* sp. by the PCR-based method described elsewhere (23, 24). Human-specific *Bacteroides* spp. have shown to be reliable indicators of this type of contamination in the water environments [10]. Based on these results, we inferred that the coprolites under study were of human origin and of sufficiently high quality to warrant further study. Moreover, the microbiological evidence in the present study also indicates the source (child vs. adult) of the coprolite samples. The presence of *Micrococcus* sp. in the Saladoid coprolites, may suggest that the samples belong to children, since several species have been associated with nurslings [11]. This is further supported by the presence of *Lactobacillus* spp., common in the feces of breast-fed children [12]. Similarly, in the Huecoid coprolite samples, the presence of bacteria belonging to the genera *Leuconostoc* suggests that the samples belong to children as well, as these bacteria are associated with maternal milk [13]. Interestingly, based on the microbial profiles, these children also consumed solid food and this accounted for the presence of bacteria commonly present in animals and plants, and pathogenic plant and edible fungi. This is also supported by the comparatively small size of the coprolites [7]. Notably, the similarity between the microbial communities in the coprolite samples of the Saladoid and Huecoid cultures was 40%. Previous studies have suggested that the intestinal microbiota between children is more dissimilar than the intestinal microbiota of adults of the same culture (5).

Our results are consistent with previous reports in which the human intestinal microbiome varies according to and is affected by diet and cultural traditions [2], [14], [15], [16]. Our report is the first to simultaneously report the bacterial, fungal and archaeal communities of human coprolites. Metagenomics studies have attempted to describe the bacterial communities in coprolites; thus, comparisons of our results with previous studies are restricted to the bacterial fraction. Human coprolites from North, Central and South America harbor Firmicutes. Coprolite samples from Central America seem to exhibit a greater diversity of bacterial communities as these harbor bacteria from the Bacteroidetes, Actinobacteria and Proteobacteria groups [1]. Although the Saladoid coprolite samples from Guayanilla and Vieques clustered, there are still differences that accounted for the separation of the samples in the MDS plots, the differences for which still need to be determined. Multivariate statistics, including PCA, MDS and cluster analysis were used to assess the ecological and diversity features of the coprolites. Ordination methods such as PCA and MDS were useful in identifying groups of individuals or samples that share characteristics in common. The utility of univariate and multivariate analyses in analyzing microbial community structure from an array of taxa or TRFs has been shown [17]. The Saladoid and Huecoid cultures exhibited significant differences in their intestinal bacterial and fungal profiles as assessed by multivariate as well as ANOSIM and SIMPER analyses. This accounted for the clustering of the Huecoid and Saladoid coprolite samples by culture; however, microbial variation within populations is very extensive, and depends on age, diet, and culture [2]. More samples would be required to fully conclude that differences between the Saladoid and Huecoid are strictly cultural and not environmental or ecological. Results would also need to be supported by mitochondrial DNA analyses, but the present study opens the opportunity to perform such analyses.

Putative taxonomic identification of bacteria and fungi (**Table 2**) was assessed by comparing the predicted TRF size from three different restriction enzymes with bacteria, archaeal and fungal databases of predicted fragment size as described by Kaplan et al (19, 25). The reliability of the taxon identification increases as the number of restriction enzymes is used. Kitts (19) and Kaplan et al. (25) propose that the use of three enzymes can provide reliable, putative taxonomic identification of principal TRFs. Coprolite samples of the Saladoid society exhibited the presence of bacteria that are commonly found in aquatic animals. Such is the case of *Vibrio* spp. (present in marine waters and in association with aquatic animals) and Actinobacteria (certain species are found in the intestines of fish) [18], supporting that the Saladoid culture included aquatic animals in their diets. The Huecoid coprolite samples were characterized by bacteria involved in cellulose degradation (*Cellulomonas* spp.) and leaf-associated bacteria (*Methylobacteria*) [19], [20]. In terms of the fungi, the Saladoid coprolite samples harbored DNA from *Debaryomyces*, a marine yeast resistant to salt concentrations of up to 24%, which has been isolated from fish [21]. Other fungal genera in the Saladoid coprolite samples included the Ascomycetes, which although is a wide group, certain species are edible. This suggests that the Saladoid culture may have included Ascomycetes in their diets, although certain species are plant pathogens and it remains a possibility that individuals ingested these fungi when consuming contaminated food or decaying vegetable matter. Other plant pathogenic fungi present in the Saladoid coprolite samples included *Melanconium* sp. and *Venturia* spp., confirming the possible ingestion of contaminated plants. From the results it appears that the Huecoid culture included fungi such as *Leucoagaricus* and *Pleurotus* spp. as part of their diets. Notably, *Pleurotus* species such as *P. ostreatus* and *P. pulmonarius* are used by some cultures around the world for anti -bacterial, -viral, inflammatory and -tumor treatment [22]. It is possible that the Huecoid culture ingested these mushrooms for medicinal purposes as well [22]. Yet, it remains to be addressed if several of the identified animal remains in the Saladoid and Huecoid sites were used for consumption and/or as pets and if these could have directly and/or indirectly influenced the intestinal microbiota.

The present study lends support to the hypothesis proposed by Chanlatte and Narganes, that the Saladoid and Huecoid cultures may be different cultures. Given that two samples were analyzed, and that it would be difficult collecting more samples due to their unique nature, results may not truly reflect the intestinal microbiota of the population, rather of a subgroup. It should be noted that the Saladoid refuse deposits in Vieques are located in the north, west and central regions of the Sorcé archeological site and most of the Huecoid deposits are located to the southern part. In addition, there is no stratigraphic superposition of the Saladoid and Huecoid cultural materials in the archeological site of Sorcé, indicating that each deposit corresponds to a specific culture. A stratigraphic superposition would indicate that a more recent culture occupied the space previously inhabited by an older culture, but this is not the case for the Saladoid and Huecoid cultures in Sorcé. The Saladoid and Huecoid sites in Vieques are separated by a distance of 15–150 m, and thus the location where the neighbor culture was established was highly accessible. This would suggest that differences between both groups would be largely cultural rather than environmental or ecological.

Contamination with exogenous microorganisms may represent a concern in coprolite studies [23]. However, T-RFLP has sufficient discriminatory power for the identification of microbes from fecal sources by comparisons with contemporary human fecal microbiota. The microbial community of coprolites was reflective of the normal human fecal flora [24] and thus lends further credence that the results obtained originated from coprolite DNA and not environmental contamination. The present study is among the few performed using T-RFLP to study microbial profiles in human coprolites. Although T-RFLP is a library-dependent method, it is less expensive than metagenomic sequencing, results are obtained within 3 to 4 days, and bacterial, fungal and archaeal analyses can be performed individually or altogether [25]. In the present study, bacteria from the groups Proteobacteria (*Vibrio* and *Desulfovibrio* spp.), Bacteroidetes, Firmicutes (*Clostridium* sp.) and Actinobacteria (*Micrococcus* and *Corynebacterium* spp.) were detected using T-RFLP and these profiles are very similar to those using a metagenomic approach in human coprolites [1]. It should also be noted that bacteria detected in the human coprolites in the present study do not correspond to those previously described in tropical soils [26]. This lends credence to the observation that bacteria detected in the coprolite samples are from a fecal origin.

### Conclusions

Our results suggest that the intestinal microbial profiles of earlier and modern cultures possess a core microbiome. This accounts for the matching of the intestinal microbial profiles of pre-Columbian cultures with those of T-RFLP databases, although specific bacterial and fungal communities accounted for differences between the coprolite samples. Based on fecal microbial community comparisons, it is apparent that the Huecoid and Saladoid cultures differ, at least in part, by the nature of their diet. When observed that these two societies virtually share the same differences were based on cultural differences. While the results are encouraging and support the two-culture hypothesis, further analyses are required to substantiate the favored hypothesis. The approach considered in the present study could be applied to characterize the intestinal microbiota of various cultures.

## Materials and Methods

### Sample Description

Coprolite samples were originally obtained by Yvonne M. Narganes-Storde and Luis Chanlatte, archeologists at the Center of Archeological Research at the University of Puerto Rico. All necessary permits were obtained for the described study, which complied with all relevant regulations. A total of five coprolites, dating 180 A.D. to 600 A.D., were analyzed (**Table 1** **)**. Four samples originated from the archeological site of Sorcé in the island of Vieques (**Figure 1**) and one, used as a control, from the archeological site of Tecla 1 in Guayanilla, in south central Puerto Rico. Two of the Sorcé samples, as well as the Guayanilla sample, were of a Saladoid origin and the remaining two samples were of a Huecoid origin.

### Sample Handling

All experiments were performed in an Ancient DNA laboratory where DNA extraction is conducted in class II hoods, earmarked for ancient DNA, exclusively. The hoods are routinely decontaminated with chlorine and PCR reactions are prepared in a DNA-free room, maintained under germicidal UV light while not in use. Sterile, DNA-free instruments were used to extract the DNA. Controls are done *ad-libitum* for the absence of extraneous DNA.

### Macromolecule Detection, DNA Extraction and PCR Amplifications

All procedures, including sample preparation, DNA extraction and PCR amplification were conducted in a laboratory earmarked for ancient DNA studies and where DNA extraction was conducted in decontaminated hoods. PCR mixtures were conducted in DNA-free rooms and physically separated from all DNA handling spaces. The exterior shell of the coprolites was removed in order to minimize environmental contamination using a sterilized brush [1]. Once the exterior shell was removed, the core of the coprolites (around 0.25 g) was extracted using aseptic techniques with gloved hands and sterile instruments in a laminar flow cabinet to minimize environmental contamination. Coprolite samples were analyzed for the presence of DNA, proteins and lipids by cytochemical staining using Acridine Orange, Fast Green, and Nile Red, respectively. Samples were reconstituted at 10 mg/mL in phosphate buffered saline. Fast Green FCF (5∶100 (v/v)), for protein staining) and Nile Red (2∶100 (v/v)), for intracellular lipid staining) was added to 100 µL of the reconstituted sample and incubated protected from light for 30 minutes at room temperature. Twenty-five µL of stained suspension was mixed with 25 µL of melted agarose (0.5% w/v) and placed on a concave microscope slide, then immediately covered with a cover-slip. Imaging was done with a confocal laser scanning microscope (CLSM) Fluoview FV1000 system equipped with an IX81 inverted microscope (Olympus, Tokyo, Japan). The observations were made with a PLAPON 60X immersion oil objective (0lympus). FCF was excited with the 633 nm HeNeR laser and Nile Red with the 488 nm AR line. Images were analyzed with the Fluoview FV1000 software (version 1.7.2.2, Olympus).

DNA was extracted using the PowerSoil® DNA Isolation Kit (Mo Bio Laboratories, Carlsbad, CA), following the manufacturer’s instructions with the exception that samples were placed in the PowerBead tubes overnight at –20°C. DNA quantity was estimated using a Qubit® 2.0 fluorometer (Life Technologies, Carlsbad, CA) and stored at –20°C until used. All PCR primers in the present study are described in **Table 7** [27], [28], [29], [30], [31]. PCR reactions for both human and dog *Bacteroides* were performed in total volumes of 50 µL and with the following reagent concentrations: 1X GoTaq® buffer (Promega Corp.), 0.4 µM dNTP (Promega Corp.), 1 µM MgCl_2_ (Promega Corp.), 2 U GoTaq® DNA polymerase (Promega Corp.), 0.5 µM HF183 or BacCan forward primer, 0.5 µM Bac708R reverse primer, and 10 ng of template DNA. PCR conditions consisted of an initial denaturation step of 95°C for 4 min, followed by 35 cycles at 95°C for 30 s, 57.5°C for 30 s, 72°C for 1 min and a final extension at 72°C for 5 min.

**Table 7:** Primers in the present study included those for human and dog Bacteroides, universal primers for the 16S rRNA of bacteria and archaea, and the ITS region of fungi.

Primers	Sequence	Direction	Target	Reference
HF183F	ATCATGAGTTCACATGTCCG	Forward	Human Bacteroides	Bernhard and Field, 2000.
BacCan	GGAGCGCAGACGGGTTTT	Forward	Dog Bacteroides	Kildare et. al., 2007
Bac708R	CAATCGGAGTTCTTCGTG	Reverse	Human and Dog Bacteroides	Bernhard and Field, 2000.
8dF	AGAGTTTGTTCMTGGCTCAG	Forward	Bacterial 16S rRNA gene	Kaplan et. al., 2001
K2R	GTATTACCGCGGCTGCTGG	Reverse	Bacterial 16S rRNA gene	Kaplan et. al., 2001
Arch21F	TTCCGGTTGATCCYGCCGGA	Forward	Archaeal 16S rRNA gene	DeLong, 1992.
Arch958R	YCCGGCGTTGAMTCCAATT	Reverse	Archaeal 16S rRNA gene	DeLong, 1992.
ITS1F	CTTGGTCATTTAGAGGAAGTAA	Forward	Fungi ITS region	Gardes and Bruns, 1993
ITS4B	TCCTCCGCTTATTGATATGC	Reverse	Fungi ITS region	Gardes and Bruns, 1993

All PCR reactions for bacteria, fungi and archaea were carried in triplicate. For the bacterial 16SrRNA gene, reactions were carried in 50 µL volumes reactions with 1X GoTaq® buffer, 0.6 µM dNTP, 3.5 µM MgCl_2_, 0.8 µg BSA (2 ml of 20 µg/mL), 2 U GoTaq®, 0.2 µM labeled primer *8dF and 0.2 µM primer K2R (10 uM) and 10 ng of DNA template. PCR conditions consisted of an initial denaturation at 94°C for 10 min, followed by 40 cycles at 94°C for 1 min, 46.5°C for 1 min, 72°C for 2 min and a final extension at 72°C for 10 min. The PCR reactions of the ITS region of fungi were carried in 50 µL volumes with 1X GoTaq*®* buffer, 0.6 µM dNTP, 2.5 µM MgCl_2_, 2 U of GoTaq® DNA polymerase, 0.2 µM of labeled ITS1F primer, 0.2 µM ITS4R primer and 10 ng template DNA. Reaction conditions consisted of an initial denaturation at 94°C for 5 min, 13 cycles of 94°C for 35 s, 55°C for 55 s and, 72°C for 45 s; 13 cycles of 94°C for 35 s, 55°C for 55 s and, 72°C for 2 min; 9 cycles of 94°C for 35 s, 55°C for 55 s and, 72°C for 3 min; followed by 72°C for 10 min. PCR products were stained using ethidium bromide (0.5 ng/L) and visualized in 1% agarose gels. For the PCR amplification of the archaeal 16S rRNA gene, reactions were performed in 50 µL with 1X GoTaq® buffer, 0.8 µM dNTP, 2.5 µM MgCl_2_, 0.8 µg BSA (2 ml of 20 µg/mL), 2 U GoTaq®, 0.2 µM labeled primer Arch21F and 0.2 µM primer Arch958R (10 uM) and 10 ng of DNA template. Reaction conditions consisted of an initial denaturation at 94°C for 10 min, 40 cycles at 94°C for 1.5 min, at 55°C for 1.5 min, 72°C for 1 min; and a final extension of 10 min at 72°C.

### Terminal Restriction Fragment (T-RFLP) Analyses

PCR products were purified using the MoBio PCR UltraClean® Kit following the manufacturer’s instructions. The fluorescently labeled amplicons (50 ng) of the bacterial and archaeal 16S rRNA gene and fungal ITS region were separately digested using two to three restriction endonucleases. Bacterial 16S rRNA gene amplicons were digested using *Dpn*II, *Hae*III and *Hha*I, fungal ITS amplicons were digested using *Aci*I, *Hae*III and *Hpa*I, and the archaeal 16S rRNA gene amplicons were digested using *Hae*III and *Hha*I. T-RFLP analyses of bacteria, archaea and fungi were conducted as described previously [25], [29]. Briefly, digestions were carried out using a thermocycler program of 37°C for 4 h and either 65°C or 80°C for 20 min. After ethanol precipitation the DNA was dissolved in 20 µL of formamide (Beckman Coulter) with 0.25 µL of 600 base pair size standard (Beckman Coulter). The fragments were separated using capillary gel electrophoresis on the CEQ8000 (Beckman Coulter). Terminal restriction fragment length in nucleotides, and TRF peak area were exported from the CEQ8000 into EXCEL (Microsoft, Seattle, WA). To standardize the data for comparison between samples, the area under each TRF peak was normalized to the total amount of DNA analyzed and expressed as parts per million (ppm). Peaks with an area of less than 5000 ppm (<0.5% of the total for that sample) were excluded from analysis to reduce noise.

### Statistical Analyses

TRF data matrices were transformed by taking the square root of the area as described previously [32]. For statistical analysis of TRF peaks, results from all enzymatic digests for each of the microbial groups were pooled into a single matrix. Sørensen's similarity index [33] was used to determine similarities in microbial community structure in each of the five coprolites [34]. Similarity matrixes were used to construct dendrograms. Additionally, the similarity matrix was analyzed with a one-way analysis of similarities (ANOSIM, Primer E software v. 6) to test the null hypothesis that association of individual TRFs with coprolites was independent of site. Global R sample statistics were computed for each comparison as described [34], [35]. Species accumulation plots were constructed to assess whether or not the sites were effectively sampled. Multidimensional scaling (MDS) plots were constructed using a similarity matrix comprised of T-RFLP coprolite results (Primer E software v. 6) [34]. The MDS plot was used to arrange samples in two-dimensional space according to their relative similarities and the BvSTEP procedure was used to select the OTUs that were the best predictors of the patterns [36]. The OTUs most responsible for the overall pattern were separated from those considered to be outliers, and separate MDS plots were made for each group. The similarity percentages-species contributions one-way analysis (SIMPER, Clarke, 1993) was used to quantify the contribution of each TRF to within-site similarity and between-site dissimilarity. Standard indices of diversity (DIVERSE), including total TRFs (S), Margalef species richness, Pielou’s evenness, Shannon diversity index (H’), and Simpson’s diversity index were calculated for all enzymes and taxa used in T-RFLP analyses (Primer E software v. 6) [34].

### Database Matching of TRF Peaks

TRFs designated by SIMPER analysis to contribute significantly to within culture similarity and/or between culture dissimilarity were assigned a putative taxonomic identification by matching predicted TRF peaks to in-house and public databases. Databases for eubacteria, archaea and fungi were created from the Ribosomal Database Project [37] and GenBank® as described previously [38].

## References

[1] Tito RY, Knights D, Metcalf J, Obregon-Tito AJ, Cleeland L, et al (2012) Insights from characterizing extinct human gut microbiomes. PLoS One 7: e51146. 23251439 10.1371/journal.pone.0051146 PMC3521025

[2] Yatsunenko T, Rey FE, Manary MJ, Trehan I, Dominguez-Bello MG, et al (2012) Human gut microbiome viewed across age and geography. Nature 486: 222–227. 22699611 10.1038/nature11053 PMC3376388

[3] Chanlatte L, Narganes I (1980) La Hueca, Vieques: nuevo complejo cultural agroalfarero en la Arqueologia Antillana. Proceedings of the 8th International Congress for Caribbean Archaeology: 501–523.

[4] Pagan-Jimenez JR (2007). De Antiguos Pueblos y Culturas Botanicas en el Puerto Rico Indigena. Oxford: Archaeopress.

[5] Pagan-Jimenez JR (2009). El Mundo Vivido por los Antiguos pobladores. San Juan, PR: Arqueología y democratización del conocimiento.

[6] Narganes-Storde YM (1982) Vertebrate faunal remains from Sorce, Vieques, Puerto Rico. Athens, GA: University of Georgia.

[7] Reinhard KJ, Bryant VM, Jr. (1992) Coprolite Analysis: A Biological Perspective on Archaeology. Papers in Natural Resources.

[8] Coleman DC, Corbin FT (1991) Introduction and Ordinary Counting as Currently Used. In: Coleman DC, Fry D, editors. Carbon Isotope Techniques. San Diego, California: Academic Press.

[9] Sobolik KD (1990) A nutritional analysis of diet as revealed in prehistoric human coprolites. The Texas Journal of Science 42.

[10] Scott TM, Rose JB, Jenkins TM, Farrah SR, Lukasik J (2002) Microbial source tracking: current methodology and future directions. Appl Environ Microbiol 68: 5796–5803. 12450798 10.1128/AEM.68.12.5796-5803.2002 PMC134426

[11] Kendall AI, Haner RC (1924) *Micrococcus ovalis* . J Infect Dis 35: 67–76.

[12] Ahrne S, Lonnermark E, Wold AE, Aberg N, Hesselmar B, et al (2005) Lactobacilli in the intestinal microbiota of Swedish infants. Microbes Infect 7: 1256–1262. 16002310 10.1016/j.micinf.2005.04.011

[13] Cabrera-Rubio R, Collado MC, Laitinen K, Salminen S, Isolauri E, et al (2012) The human milk microbiome changes over lactation and is shaped by maternal weight and mode of delivery. Am J Clin Nutr 96: 544–551. 22836031 10.3945/ajcn.112.037382

[14] Li M, Wang B, Zhang M, Rantalainen M, Wang S, et al (2008) Symbiotic gut microbes modulate human metabolic phenotypes. Proc Natl Acad Sci U S A 105: 2117–2122. 18252821 10.1073/pnas.0712038105 PMC2538887

[15] Nelson KE, Weinstock GM, Highlander SK, Worley KC, Creasy HH, et al (2010) A catalog of reference genomes from the human microbiome. Science 328: 994–999. 20489017 10.1126/science.1183605 PMC2940224

[16] Mueller S, Saunier K, Hanisch C, Norin E, Alm L, et al (2006) Differences in fecal microbiota in different European study populations in relation to age, gender, and country: a cross-sectional study. Appl Environ Microbiol 72: 1027–1033. 16461645 10.1128/AEM.72.2.1027-1033.2006 PMC1392899

[17] Frey JC, Pell AN, Berthiaume R, Lapierre H, Lee S, et al (2010) Comparative studies of microbial populations in the rumen, duodenum, ileum and faeces of lactating dairy cows. J Appl Microbiol 108: 1982–1993. 19863686 10.1111/j.1365-2672.2009.04602.x

[18] Hamada M, Iino T, Tamura T, Iwami T, Harayama S, et al (2009) *Serinibacter salmoneus* gen. nov., sp. nov., an actinobacterium isolated from the intestinal tract of a fish, and emended descriptions of the families Beutenbergiaceae and Bogoriellaceae. Int J Syst Evol Microbiol 59: 2809–2814. 19628613 10.1099/ijs.0.011106-0

[19] Kutschera U (2007) Plant-associated methylobacteria as co-evolved phytosymbionts: a hypothesis. Plant Signal Behav 2: 74–78. 19516971 10.4161/psb.2.2.4073 PMC2633902

[20] Dermoun Z, Belaich JP (1985) Microcalorimetric study of cellulose degradation by *Cellulomonas* uda ATCC 21399. Biotechnol Bioeng 27: 1005–1011. 18553770 10.1002/bit.260270711

[21] Reyes-Becerril M, Salinas I, Cuesta A, Meseguer J, Tovar-Ramirez D, et al (2008) Oral delivery of live yeast Debaryomyces hansenii modulates the main innate immune parameters and the expression of immune-relevant genes in the gilthead seabream (*Sparus aurata* L.). Fish Shellfish Immunol 25: 731–739. 19004644 10.1016/j.fsi.2008.02.010

[22] Wasser MP, Weis AL (1999) Medicinal Properties of Substances Occurring in Higher Basidiornycetes Mushrooms: Current Perspectives (Review). International journal of Medicinal Mushrooms 1: 31–62.

[23] Wood JR, Wilmshurst JM, Wagstaff SJ, Worthy TH, Rawlence NJ, et al (2012) High-resolution coproecology: using coprolites to reconstruct the habits and habitats of New Zealand's extinct upland moa (Megalapteryx didinus). PLoS One 7: e40025. 22768206 10.1371/journal.pone.0040025 PMC3386916

[24] Holdeman LV, Good IJ, Moore WE (1976) Human fecal flora: variation in bacterial composition within individuals and a possible effect of emotional stress. Appl Environ Microbiol 31: 359–375. 938032 10.1128/aem.31.3.359-375.1976 PMC169780

[25] Kitts CL (2001) Terminal restriction fragment patterns: a tool for comparing microbial communities and assessing community dynamics. Curr Issues Intest Microbiol 2: 17–25. 11709853

[26] DeAngelis KM, Gladden JM, Allgaier M, D’haeseleer P, Fortney JL, et al (2010) Strategies for Enhancing the Effectiveness of Metagenomic-based Enzyme Discovery in Lignocellulolytic Microbial Communities. Bioenerg Res 3: 146–158.

[27] Bernhard AE, Field KG (2000) A PCR assay To discriminate human and ruminant feces on the basis of host differences in *Bacteroides-Prevotella* genes encoding 16S rRNA. Appl Environ Microbiol 66: 4571–4574. 11010920 10.1128/aem.66.10.4571-4574.2000 PMC92346

[28] Kildare BJ, Leutenegger CM, McSwain BS, Bambic DG, Rajal VB, et al (2007) 16S rRNA-based assays for quantitative detection of universal, human-, cow-, and dog-specific fecal Bacteroidales: a Bayesian approach. Water Res 41: 3701–3715. 17644149 10.1016/j.watres.2007.06.037

[29] Kaplan CW, Astaire JC, Sanders ME, Reddy BS, Kitts CL (2001) 16S ribosomal DNA terminal restriction fragment pattern analysis of bacterial communities in feces of rats fed *Lactobacillus acidophilus* NCFM. Appl Environ Microbiol 67: 1935–1939. 11282651 10.1128/AEM.67.4.1935-1939.2001 PMC92815

[30] DeLong EF (1992) Archaea in coastal marine environments. Proc Natl Acad Sci U S A 89: 5685–5689. 1608980 10.1073/pnas.89.12.5685 PMC49357

[31] Bruns TD, Gardes M (1993) Molecular tools for the identification of ectomycorrhizal fungi–taxon-specific oligonucleotide probes for suilloid fungi. Mol Ecol 2: 233–242. 7513242 10.1111/j.1365-294x.1993.tb00013.x

[32] Blackwood CB, Marsh T, Kim SH, Paul EA (2003) Terminal restriction fragment length polymorphism data analysis for quantitative comparison of microbial communities. Appl Environ Microbiol 69: 926–932. 12571013 10.1128/AEM.69.2.926-932.2003 PMC143601

[33] Sorensen T (1948) A method of establishing groups of equal amplitude in plant sociology based on similarity of species and its application to analyses of the vegetation on Danish commons. Biologiske Skrifter 5: 1–34.

[34] Clarke KR, Gorley RN (2006) PRIMER v6: User Manual/Tutorial. PRIMER-E, Plymouth.

[35] Clarke KR, Warwick RM (2001) Change in marine communities: an approach to statistical analysis and interpretation: PRIMER-E, Plymouth.

[36] Clarke KR, Warwick RM (1994) Similarity-based testing for community pattern: the 2-way layout with no replication. Mar Biol 118: 167–176.

[37] Cole JR, Wang Q, Cardenas E, Fish J, Chai B, et al (2009) The Ribosomal Database Project: improved alignments and new tools for rRNA analysis. Nucleic Acids Res 37: D141–145. 19004872 10.1093/nar/gkn879 PMC2686447

[38] Kaplan CW, Kitts CL (2004) Bacterial succession in a petroleum land treatment unit. Appl Environ Microbiol 70: 1777–1786. 15006804 10.1128/AEM.70.3.1777-1786.2004 PMC368334

